# Evaluation of Outflow Structures *In Vivo* after the Phacocanaloplasty

**DOI:** 10.1155/2016/4519846

**Published:** 2016-07-19

**Authors:** Daiva Paulaviciute-Baikstiene, Renata Vaiciuliene, Vytautas Jasinskas, Ingrida Januleviciene

**Affiliations:** Department of Ophthalmology, Lithuanian University of Health Sciences, Eiveniu Street 2, LT-50009 Kaunas, Lithuania

## Abstract

*Purpose*. To evaluate the* in vivo* changes in Schlemm's canal (SC) and the trabecular meshwork (TM) in patients with primary open-angle glaucoma (POAG) after phacocanaloplasty using anterior segment optical coherence tomography (AS-OCT).* Methods*. Ten eyes of nine patients with POAG (6 men and 3 women) who underwent phacocanaloplasty. Preoperative and postoperative visual acuity (VA), intraocular pressure (IOP), and use of glaucoma medications were evaluated. The main outcome measures were the area of SC and TM thickness assessed using AS-OCT before and 12 months after surgery.* Results*. We found statistically significant reduction in IOP (from 26.4 (8.6) mmHg to 12.9 (2.5) (*p* < 0.05) mmHg), increase in VA from 0.7 (0.4) to 0.9 (0.2), and decrease in glaucoma medication from 2.6 (1.2) to 1.1 (1.3) at 12 months postoperatively. There was a significant increase in the SC area (3081.7 (842.8) *μ*m^2^ versus 5098.8 (1190.5) *μ*m^2^, *p* < 0.001) and a decrease in mean TM thickness (91.2 (18.6) *μ*m versus 81.3 (15.1) *μ*m, *p* = 0.001) after surgery. We found negative correlations between SC area and IOP before surgery (*r* = −0.67, *p* = 0.03) and also between SC area before and IOP reduction 12 months after the phacocanaloplasty (*r* = −0.80, *p* = 0.005).* Conclusions*. Our results showed statistically significant dilation of SC area and reduction of TM thickness after phacocanaloplasty in POAG patients. The degree of SC expansion was related to the IOP decrease.

## 1. Introduction

Elevated intraocular pressure (IOP) is the only modifiable risk factor for the presence and progression of glaucoma [1]. The conventional drainage route of aqueous humor occurs via the trabecular meshwork (TM), Schlemm's canal (SC), collector channels, and aqueous veins and then streams into episcleral venous circulation. Appropriate secretion and regulation of aqueous humor are essential for normal eye function [2].

SC plays an important role in regulating IOP in the human eye [3, 4]. Dimensions of SC may correlate with the IOP fluctuations [5]. When topical glaucoma medication treatment is ineffective Schlemm's canal based nonperforated glaucoma surgery is considered to be the most effective method to lower IOP [6]. Canaloplasty is an advanced interventional glaucoma treatment. It has gained increasing popularity as a surgical procedure by attempting to stimulate the natural outflow pathway and to be independent of filtering blebs [7]. The point of canaloplasty effect is circumferential dilation and suture tensioning of SC inner wall, after which SC is permanently enlarged and better access to the collector channels is achieved [8].

In a small number of eyes, this surgery could be unsuccessful because of a nonreversible disruption of collector channels or other outflow pathways that cannot be expanded due to anatomical factors [9]. Technological advances like anterior segment optical coherence tomography (AS-OCT) with high axial resolution allow us to observe* in vivo* structural and pathophysiological changes in SC and the TM thickness [10]. Only a few studies assessed the anatomical changes of SC and TM before and after a nonpenetrating surgical procedure. The purpose of the current study was to evaluate and to compare SC and TM parameters before and one year after phacocanaloplasty in patients with primary open-angle glaucoma (POAG) using AS-OCT. Main outcome measurements included SC area and TM thickness with respect to IOP as a secondary variable.

## 2. Materials and Methods

All study procedures were carried out according to the Declaration of Helsinki, and the study protocol was approved by the Lithuanian University of Health Sciences Review Board. Study objectives and methods were explained to all the subjects prior to the examination. Written informed consent was obtained from all the patients.

The inclusion criteria for surgical cataract and glaucoma treatment were POAG patients over 18 years with medically uncontrolled IOP, progressing glaucoma with diffuse and/or subcortical and/or nuclear lens opacities that influenced visual acuity or visual function that no longer met patients' needs. One patient had an allergy to all IOP-lowering medications. Pregnant or nursing women, patients with uncontrolled systemic diseases, previous ocular surgery, secondary glaucoma, and congenital glaucoma, and patients with a history of other eye diseases or trauma were excluded from the study.

Ophthalmic examination consisted of Snellen best-corrected visual acuity (BCVA), IOP (Goldmann tonometer—an average of 3 separate IOP measurements was taken), slit-lamp biomicroscopy, gonioscopy (VOLK, Three-Mirror Lens), fundus examination measurements, visual field testing (24-2 SITA-Standard strategy; Humphrey Standard Achromatic Perimetry), and AS-OCT (Nidek, RS-3000, Advance, Japan) before and 12 months after the phacocanaloplasty. Patients were examined at the same time of the day to avoid fluctuations.

### 2.1. Surgical Procedure

Phacocanaloplasty is a relatively new nonpenetrating surgical procedure [9]. The conjunctiva was dissected at the limbus in the superior medial segment. A 5.0 × 5.0 mm parabolic shape superficial scleral flap was created including about one-third of superficial scleral thickness. The second scleral flap was of the same shape but slightly smaller than the superficial one and involved almost all remaining scleral thickness. The flap was dissected to the limbal area until SC was localized. Later continuous curvilinear capsulorhexis, standard phacoemulsification, and endocapsular foldable intraocular lens (IOL) implantation were performed. After cataract surgery, SC was unroofed and remnants of corneal stroma were striped from the Descemet membrane (DM) about 0.5 mm towards the center of the cornea. Created flap was excised performing a window of DM. Ostia of SC were dilated with Healon GV® with a micro cannula. A flexible microcatheter (iTrack-250A, iScience) was introduced into the ostia of SC. After complete 360° passing of the iTrack® a Prolene Suture (10-0) was fixed to the distal tip of the microcatheter and looped through the canal. At the same time, SC was filled and dilated with Healon GV. The suture was tightened to stretch the inner wall of SC and the TM circumferentially. The distension of the canal was observed clinically by the inward movement of the suture and inner wall of exposed SC at the surgical access area. The suture was tightened under tension on a soft eye and tied in order to stretch the TM circumferentially and to open the SC when IOP returned to normal. The superficial flap was closed and sutured watertight with 10-0 vicryl sutures.

All surgical procedures were performed by the same surgeon V. J.

### 2.2. SC and TM Measurements Using Optical Coherence Tomography

For SC and TM scanning, all subjects underwent spectral-domain AS-OCT imaging (Nidek, RS-3000, Advance, Japan). Cross-sectional images are captured with the optical interferometer using an infrared light source with a wavelength of 880 nm. Scans centered on the pupil and were obtained using the standard anterior segment single-scan protocol in the horizontal meridian, at the 3 and 9 o'clock position. Baseline SC scanning was performed one day before phacocanaloplasty. Follow-up angle scanning was carried out twelve months after surgery. The SC was observable in all ten eyes which were included in the study. Based on image quality three images were chosen for final analysis.

### 2.3. Image Analysis

The AS-OCT images of SC were imported in ImageJ (ImageJ v1.50b, NIH, https://imagej.nih.gov/ij/) for analysis after they were enhanced with the adaptive compensation algorithm. Contrast and magnification were adjusted to maximize visualization. Scans with poor resolution and/or nonvisible SC were excluded. According to the previous studies, SC was defined as observable when the thin, black, lucent space was found outside of the TM on the AS-OCT images [11].

**Figure 1:**
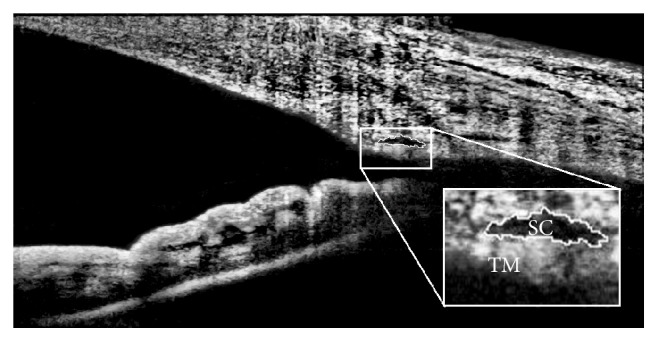
Visualization of outflow structures (SC, TM) with AS-OCT. The image was adjusted to maximize visualization with ImageJ program.

The SC area was drawn freehand and represented the area surrounded by the outline of SC and then was measured manually by a masked operator (Figure 1). The area of SC in each site was recorded as the arithmetic mean of measurements from three images. TM thickness was measured below the SC from the anterior to the posterior end point of SC. The mean of the nasal and temporal SC and TM was used in the analysis.

### 2.4. Statistical Analysis

Statistical analysis was performed using SPSS version 20.0 for Windows (IBM Corporation, Armonk, NY, USA). The level of significance *p* ≤ 0.05 was considered significant. Preoperative and postoperative data were analysed with the Wilcoxon test. Relations between data were analysed using Spearman's correlation.

## 3. Results

The mean age of participants was 51.3 (14.7) years; 3 patients were women and 6 were men (totally 10 eyes). Mean glaucoma duration was 7 (5.1) years (min 1 and max 18). The visual field mean deviation was −9.6 (10.9) dB and we found no statistically significant changes one year after surgery. Preoperative and postoperative data are shown in Table 1.

After phacocanaloplasty surgery IOP decreased statistically significantly to 12.9 (2.5) (*p* < 0.05) mmHg (or 51.1% from preoperative value). Nine eyes were under medical treatment before surgery (min 0 and max 4) and four eyes after surgery (min 0 and max 3) (*p* < 0.05).

### 3.1. Changes in Anterior Chamber Angle Morphology after the Phacocanaloplasty

**Figure 2:**
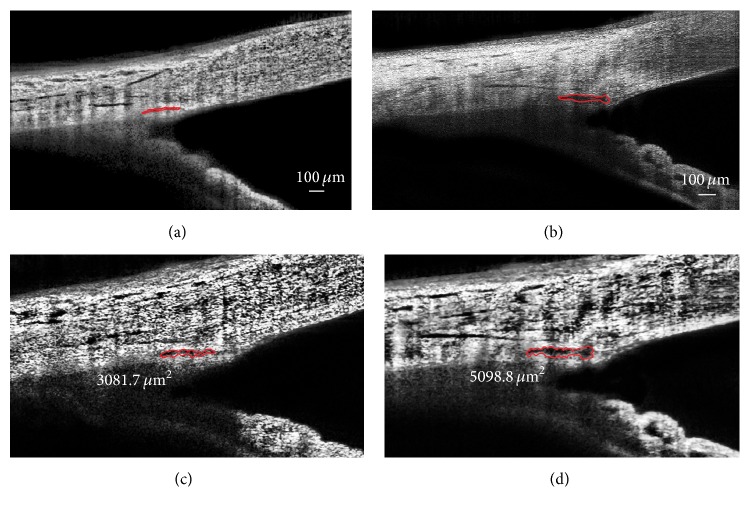
Anatomical changes of SC before ((a), (c)) and after ((b), (d)) phacocanaloplasty. Visualization was maximized by adjusting contrast and magnification using ImageJ program ((c), before surgery, and (d), after surgery).

There was a statistically significant increase in the SC area at the follow-up examination compared with the baseline value (SC area: 3081.7 (842.8) *μ*m^2^ versus 5098.8 (1190.5) *μ*m^2^, *p* < 0.001) (Figure 2). Interestingly, the mean TM thickness decreased statistically significantly after surgical treatment (91.2 (18.6) *μ*m versus 81.3 (15.1) *μ*m, *p* = 0.001).

**Figure 3:**
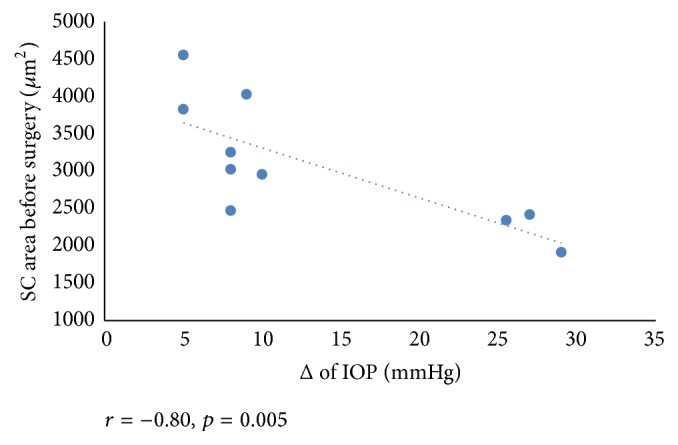
Scattergram showing the relationship between the SC area before surgery and the reduction of IOP.

**Figure 4:**
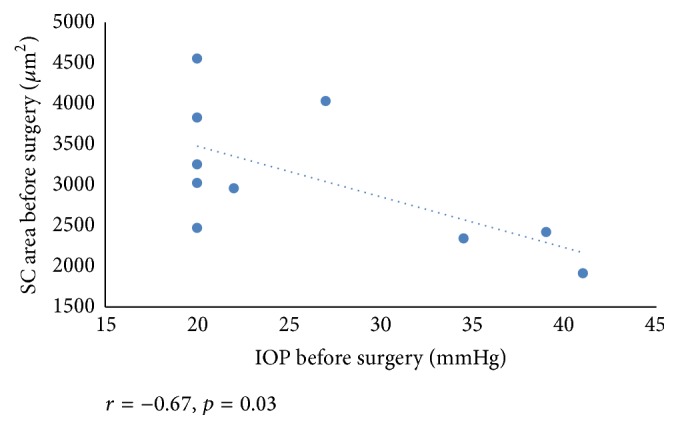
Scattergram showing the relationship between the SC area and IOP before surgery.

**Figure 5:**
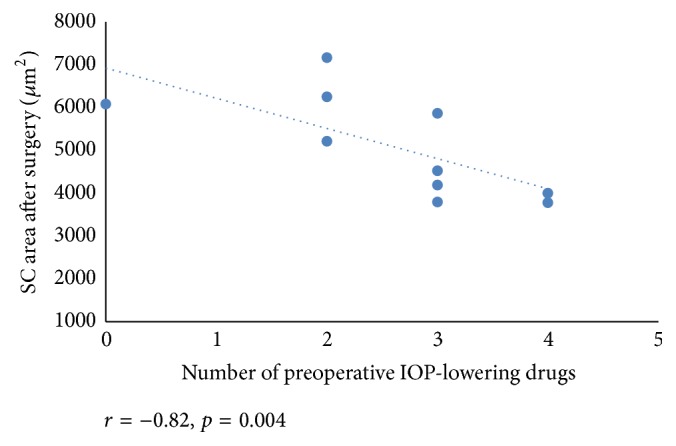
Scattergram showing the relationship between the SC area after surgery and glaucoma medication before surgery.

We found a negative correlation between reduction in IOP at 12 months after baseline and the SC area before surgery (*r* = −0.80, *p* = 0.005) (Figure 3). Also, mean SC area negatively correlated with IOP before surgery (*r* = −0.67, *p* = 0.03) (Figure 4). We found a significant correlation between a preoperative number of glaucoma medications and SC area after surgery (*r* = −0.82, *p* = 0.004) (Figure 5).

## 4. Discussion

The current study investigated changes in SC and TM as two main structures of the conventional outflow tract after the phacocanaloplasty. It is believed that IOP reduction after canaloplasty is achieved by opening previously nonfunctional areas of the outflow system and alleviating the natural aqueous drainage system through collector channels and aqueous veins [12]. The decrease of IOP after effective canaloplasty is usually assigned to the successful plasticity of the SC [13, 14].

However, earlier conclusions about SC and the TM were made* in vitro*. Technological advances (AS-OCT, UBM) let us* in vivo* evaluate outflow structures associated with phacocanaloplasty [14, 15]. AS-OCT offers a high-resolution imaging of superficial conjunctival areas, SC, and TM after nonpenetrating glaucoma surgery, whereas with UBM it is easier to detect deeper structures such as intracanal sutures and scleral lakes [14].

To our knowledge, only a few original studies aimed to assess the anatomical changes of SC after the canaloplasty. Usui et al. reported that SC area was enlarged after the pseudo viscocanalostomy in enucleated human eye. These results were supported by histologic sections [16]. Fuest and colleagues examined a total of twelve POAG patients with spectral-domain AS-OCT and UBM and found enlargement of the SC height and width three months after the nonpenetrating glaucoma surgery. They reported that the collapse in SC occurred mainly because of a reduction in height in glaucomatous eyes [14]. In the present study, we evaluated SC area; compared to Fuest different AS-OCT system was used. We were focused on the late postoperative data. Our results demonstrated the distension of the SC area one year postoperatively with sufficiently low IOP. Fuest et al. identified that dilation of SC negatively correlated with the IOP after surgery. We found a strong negative correlation between the SC area before surgery and the reduction of IOP. Studies carried out by other authors reported that a strong correlation exists between the outflow capacity and SC dimensions [5, 10, 15, 17].

Studies of single point SC measurements from healthy and POAG patients have established that SC area in healthy patients was significantly larger than in POAG patients [5, 10, 15, 18, 19]. Kagemann et al. in their study made by high-density OCT showed that acute IOP elevations in healthy eyes with open angles resulted in a reduced SC cross-sectional area [20]. They subjectively explained that compression of SC is possibly due to a movement of the inner wall towards the outer and might be a result of elevated IOP. Irshad et al. established that the diameter of SC was smaller in eyes with previous glaucoma surgery compared with eyes without glaucoma surgery [21]. Furthermore, Kagemann and colleagues obtained that SC area varies between 4064 and 7164 *μ*m^2^ [11, 20, 22–24]. Our results analysed and estimated with enough new ImageJ program showed that average of SC area was 3081.7 (842.8) *μ*m^2^ before and 5098.8 (1190.5) *μ*m^2^ after surgery. It is important to mention that the results of anterior segment structures performed with different visualization methods (UBM or AS-OST) or systems from different manufacturers may vary.

According to the previous studies, TM also could be visualized and measured* in vivo*. Filla with colleagues in a prospective study found that increased extracellular matrix expression and deposits in the TM resulted in an increase in IOP [25]. Johnstone and Grant reported that elevated IOP may induce the TM compression [26]. TM thickness measurements were performed with UBM in Yan et al. study. Authors noticed that TM thickness was smaller in POAG patients compared to healthy controls (103.9 (11.1) versus 88.3 (13.2) *μ*m). They also found a negative correlation with IOP [15]. In the present study, we measured TM thickness with AS-OCT below the SC and compared preoperative and postoperative results. We revealed that TM thickness decreased after expansion of the SC area when compared to presurgical values. To the best of our knowledge, this is the first report of the measurement of TM thickness in living human individuals after phacocanaloplasty.

Recent studies revealed a significant decrease in IOP by approximately 40% with a reduction of medications from 2.3 to 1.0 [4, 27–29] and less surgical complications in canaloplasty than in trabeculectomy [30–34]. A number of retrospective and prospective studies found better results concerning IOP reduction in combined surgical (phacocanaloplasty) cases compared with canaloplasty [32, 33]. The present study results demonstrated a significant correlation between a number of preoperative IOP-lowering drugs and SC area after surgery. Our results suggest that phacocanaloplasty surgery has been shown to be an effective and safe method to reduce IOP (51.1% from preoperative value) and a number of medications (from 2.6 to 1.1) in POAG patients.

Our study has some limitations: relatively small sample size which made it difficult to investigate SC and TM morphology at different stages of POAG. Our AS-OCT system did not have a tracking system for the exact location finding. We have followed a constant AS-OCT imaging protocol which contained scanning of specific sites (at 3 and 9 o'clock). The arithmetic mean of SC area was obtained from three images of the best quality. We have scanned anterior segment horizontally leaving the superior and inferior limbus zones not evaluated. This was done to prevent interference from the eyelids. For that reason, data may not represent overall circumferential changes in these outflow structures (SC and TM). Future studies with longer follow-up periods and a larger group of patients could help to understand the long-term effects and success rates of phacocanaloplasty.

## 5. Conclusions

Phacocanaloplasty is an effective treatment option for patients with cataract and POAG. SC and TM can be noninvasively imaged in living human eyes by AS-OCT. Our results showed statistically significant dilation of SC area and reduction of TM thickness after phacocanaloplasty. The results imply a negative correlation of the expansion of SC with the IOP-lowering effect after surgery.

## Figures and Tables

**Table 1:** Comparison between preoperative and postoperative parameters.

	Preoperative	Postoperative
BCVA	0.7 (0.4)	0.9 (0.2)
IOP, mmHg	26.4 (8.6)	12.9 (2.5)^*∗*^
MD, dB	−9.6 (10.9)	−10.53 (10.4)
Number of IOP-lowering drugs	2.6 (1.2)	1.1 (1.3)^*∗*^

BCVA: best corrected visual acuity; IOP: intraocular pressure; MD: mean deviation.

Values are shown in mean (SD), ^*∗*^ *p* < 0.05, based on Wilcoxon test.

## References

[1] Caprioli J., Coleman A. L. (2008). Intraocular pressure fluctuation. A risk factor for visual field progression at low intraocular pressures in the advanced glaucoma intervention study. *Ophthalmology*.

[2] Paulavičiute-Baikstiene D., Baršauskaite R., Janulevičiene I. (2013). New insights into pathophysiological mechanisms regulating conventional aqueous humor outflow. *Medicina*.

[3] Dautriche C. N., Tian Y., Xie Y., Sharfstein S. T. (2015). A closer look at Schlemm's canal cell physiology: implications for biomimetics. *Journal of Functional Biomaterials*.

[4] Bull H., Von Wolff K., Körber N., Tetz M. (2011). Three-year canaloplasty outcomes for the treatment of open-angle glaucoma: European Study Results. *Graefe's Archive for Clinical and Experimental Ophthalmology*.

[5] Allingham R. R., De Kater A. W., Ethier C. R. (1996). Schlemm's canal and primary open angle glaucoma: correlation between Schlemm's canal dimensions and outflow facility. *Experimental Eye Research*.

[6] Khaimi M. A. (2015). Canaloplasty: a minimally invasive and maximally effective glaucoma treatment. *Journal of Ophthalmology*.

[7] Klink T., Panidou E., Kanzow-Terai B., Klink J., Schlunck G., Grehn F. J. (2012). Are there filtering blebs after canaloplasty?. *Journal of Glaucoma*.

[8] Grieshaber M. C. (2012). Ab externo Schlemm's canal surgery: viscocanalostomy and canaloplasty. *Developments in Ophthalmology*.

[9] Brusini P. (2014). Canaloplasty in open-angle glaucoma surgery: a four-year follow-up. *The Scientific World Journal*.

[10] Hong J., Xu J., Wei A. (2013). Spectral-domain optical coherence tomographic assessment of Schlemm's canal in Chinese subjects with primary open-angle glaucoma. *Ophthalmology*.

[11] Kagemann L., Nevins J. E., Jan N.-J. (2014). Characterisation of Schlemm's canal cross-sectional area. *British Journal of Ophthalmology*.

[12] Quaranta L., Biagioli E., Riva I. (2014). Effect of trabeculectomy and canaloplasty on intra-ocular pressure modifications after postural changes in open-angle glaucoma. *Acta Ophthalmologica*.

[13] Grieshaber M. C., Pienaar A., Olivier J., Stegmann R. (2010). Clinical evaluation of the aqueous outflow system in primary open-angle glaucoma for canaloplasty. *Investigative Ophthalmology & Visual Science*.

[14] Fuest M., Kuerten D., Koch E. (2015). Evaluation of early anatomical changes following canaloplasty with anterior segment spectral-domain optical coherence tomography and ultrasound biomicroscopy. *Acta Ophthalmologica*.

[15] Yan X., Li M., Chen Z., Zhu Y., Song Y., Zhang H. (2016). Schlemm's canal and trabecular meshwork in eyes with primary open angle glaucoma: a comparative study using high-frequency ultrasound biomicroscopy. *PLoS ONE*.

[16] Usui T., Tomidokoro A., Mishima K. (2011). Identification of Schlemm's canal and its surrounding tissues by anterior segment fourier domain optical coherence tomography. *Investigative Ophthalmology & Visual Science*.

[17] Allingham R. R., De Kater A. W., Ethier C. R., Anderson P. J., Hertzmark E., Epstein D. L. (1992). The relationship between pore density and outflow facility in human eyes. *Investigative Ophthalmology and Visual Science*.

[18] Lütjen-Drecoll E., Shimizu T., Rohrbach M., Rohen J. W. (1986). Quantitative analysis of ‘plaque material’ in the inner- and outer wall of Schlemm's canal in normal- and glaucomatous eyes. *Experimental Eye Research*.

[19] Wang F., Shi G., Li X. (2012). Comparison of Schlemm's canal's biological parameters in primary open-angle glaucoma and normal human eyes with swept source optical. *Journal of Biomedical Optics*.

[20] Kagemann L., Wang B., Wollstein G. (2014). IOP elevation reduces schlemm's canal cross-sectional area. *Investigative Ophthalmology and Visual Science*.

[21] Irshad F. A., Mayfield M. S., Zurakowski D., Ayyala R. S. (2010). Variation in Schlemm's canal diameter and location by ultrasound biomicroscopy. *Ophthalmology*.

[22] Kagemann L., Wollstein G., Ishikawa H. (2010). Identification and assessment of Schlemm's canal by spectral-domain optical coherence tomography. *Investigative Ophthalmology and Visual Science*.

[23] Kagemann L., Wollstein G., Ishikawa H. (2012). Visualization of the conventional outflow pathway in the living human eye. *Ophthalmology*.

[24] Kagemann L., Wollstein G., Ishikawa H. (2011). 3D visualization of aqueous humor outflow structures in-situ in humans. *Experimental Eye Research*.

[25] Filla M. S., Schwinn M. K., Sheibani N., Kaufman P. L., Peters D. M. (2009). Regulation of cross-linked actin network (CLAN) formation in human trabecular meshwork (HTM) cells by convergence of distinct *β*1 and *β*3 integrin pathways. *Investigative Ophthalmology & Visual Science*.

[26] Johnstone M. A., Grant W. M. (1973). Pressure-dependent changes in structures of the aqueous outflow system of human and monkey eyes. *American Journal of Ophthalmology*.

[27] Matthaei M., Steinberg J., Wiermann A., Richard G., Klemm M. (2011). Canaloplasty: a new alternative in non-penetrating glaucoma surgery. *Der Ophthalmologe*.

[28] Ayyala R. S., Chaudhry A. L., Okogbaa C. B., Zurakowski D. (2011). Comparison of surgical outcomes between canaloplasty and trabeculectomy at 12 months' follow-up. *Ophthalmology*.

[29] Fujita K., Kitagawa K., Ueta Y., Nakamura T., Miyakoshi A., Hayashi A. (2011). Short-term results of canaloplasty surgery for primary open-angle glaucoma in Japanese patients. *Case Reports in Ophthalmology*.

[30] Lewis R. A., von Wolff K., Tetz M. (2007). Canaloplasty: circumferential viscodilation and tensioning of Schlemm's canal using a flexible microcatheter for the treatment of open-angle glaucoma in adults: interim clinical study analysis. *Journal of Cataract and Refractive Surgery*.

[31] Lewis R. A., von Wolff K., Tetz M. (2009). Canaloplasty: circumferential viscodilation and tensioning of Schlemm canal using a flexible microcatheter for the treatment of open-angle glaucoma in adults: two-year interim clinical study results. *Journal of Cataract & Refractive Surgery*.

[32] Shingleton B., Tetz M., Korber N. (2008). Circumferential viscodilation and tensioning of Schlemm canal (canaloplasty) with temporal clear corneal phacoemulsification cataract surgery for open-angle glaucoma and visually significant cataract: one-year results. *Journal of Cataract and Refractive Surgery*.

[33] Grieshaber M. C., Pienaar A., Olivier J., Stegmann R. (2010). Canaloplasty for primary open-angle glaucoma: long-term outcome. *The British Journal of Ophthalmology*.

[34] Matlach J., Dhillon C., Hain J., Schlunck G., Grehn F., Klink T. (2015). Trabeculectomy versus canaloplasty (TVC study) in the treatment of patients with open-angle glaucoma: a prospective randomized clinical trial. *Acta Ophthalmologica*.

